# A novel pyroptosis-related lncRNA prognostic signature associated with the immune microenvironment in lung squamous cell carcinoma

**DOI:** 10.1186/s12885-022-09790-z

**Published:** 2022-06-23

**Authors:** Wei Zhou, Wenxiong Zhang

**Affiliations:** 1 Department of Thoracic Surgery, The Second Affiliated Hospital of Nanchang University, 1 Minde Road, 330006 Nanchang, China; 2 Jiangxi medical college, Nanchang University, 330006 Nanchang, China

**Keywords:** Lung squamous cell carcinoma, Pyroptosis, Long non-coding RNA, Prognostic signature, Immunotherapy

## Abstract

**Background:**

A growing body of evidence suggests that pyroptosis-related lncRNAs (PRncRNAs) are associated with the prognoses of tumor patients and their tumor immune microenvironments. However, the function of PRlncRNAs in lung squamous cell carcinoma (LUSC) remains unclear.

**Methods:**

We downloaded the transcriptome and clinical information of 551 LUSC samples from the The Cancer Genome Atlas (TCGA) database and randomly separated patients with complete information into two cohorts. Based on the training cohort, we developed a pyroptosis-related signature. We then examined the signature in the test cohort and all retained patients. We also clustered two risk groups in each cohort according to the signature and performed survival analysis, functional analysis, tumor immune microenvironment analysis and drug sensitivity analysis.

**Results:**

A prognostic signature containing five PRlncRNAs (AP001189.1, PICART1, LINC02555, AC010422.4, and AL606469.1) was developed. A principal component analysis (PCA) indicated better differentiation between patients with different risk scores. Kaplan–Meier (K–M) analysis demonstrated poorer survival among patients with higher risk scores (*P* < 0.001). A receiver operating characteristic (ROC) curve analysis provided evidence confirming the accuracy of the signature, and univariate (*p* = 0.005) and multivariate (*p* = 0.008) Cox regression analyses confirmed the independent value of the risk score in prognoses. Clinical subgroup validation indicated that the signature was more suitable for patients with early-stage LUSC. We also created a nomogram to increase the accuracy of the prediction. Moreover, functional analysis revealed that pathways related to tumor development and pyroptosis were enriched in the high-risk group. Furthermore, the prognostic signature was proven to be a predictor of sensitivity to immunotherapy and chemotherapy.

**Conclusions:**

We developed a novel pyroptosis-associated signature with independent value for the prognosis of LUSC patients. PRlncRNAs are closely associated with the tumor immune microenvironment in LUSC and might offer new directions for immunotherapy.

**Supplementary Information:**

The online version contains supplementary material available at 10.1186/s12885-022-09790-z.

## Introduction

Lung cancer (LC) continues to maintain high lethality and mortality rates, corresponding to approximately 1.8 million deaths and 2.1 million emerging patients per year [1, 2]. As an important subtype of LC, lung squamous cell carcinoma (LUSC) accounts for approximately 30% of LC cases and is thus second only to lung adenocarcinoma (LUAD) [3]. Over the past few years, emerging biomarkers have increased the effectiveness of LUAD treatment, but little progress has been made regarding LUSC [4]. Even worse, the prognostic assessments of LUSC patients based on available clinicopathological risk factors remain unsatisfactory, and patients with identical clinicopathological characteristics often have different prognostic outcomes, which makes the prediction of prognoses challenging [5]. Therefore, a novel prognostic signature is needed to increase the accuracy of the prediction of the prognoses of LUSC patients and to more feasibly provide targeted therapy.

Pyroptosis, which is a novel form of programmed cell death, is characterized by inflammasome-dependent cell swelling and lysis [6]. Pyroptosis affects tumor development through its close association with the tumor microenvironment. For instance, natural killer (NK) cells and cytotoxic T lymphocytes promote pyroptosis by releasing granzyme to cleave GSDMB and GSDME, and thereby inhibiting tumor progression [7, 8]. Immune checkpoint inhibitors are an effective immunotherapy option due to regulating the immune microenvironment to maintain tumor growth but are applicable in only a few patients with LUSC [9]. The in-depth study of pyroptosis provides possible strategies to improve the immunotherapeutic efficacy by inducing pyroptosis and regulating the tumor immune microenvironment to convert some cold tumors into hot tumors [10]. However, the connection between the tumor immune microenvironment and pyroptosis in LUSC remains unclear.

Long noncoding RNAs (lncRNAs), which lack the potential to encode proteins, are a type of RNA with a length of more than 200 nucleotide sequences. [11]. A growing body of evidence shows the important functions of lncRNAs in tumorigenesis and metastasis [12, 13]. Studies have demonstrated that lncRNAs are closely associated with pyroptosis mechanisms, which implies that lncRNAs can potentially be used as biomarkers for early prognoses and targeted treatments [14]. Increasingly, prognostic signatures based on pyroptosis-related lncRNAs (PRlncRNAs) have been increasingly constructed for gliomas, colon cancer, and other cancers [15, 16]. However, there remains lack of PRlncRNA signatures for LUSC.

In this study, we screened the most valuable PRlncRNAs to construct a prognostic signature for patients with LUSC and validated its predictive value. Additionally, we investigated the role of pyroptosis in the tumor immune microenvironment in LUSC, with the aim of providing new insights into LUSC immunotherapy.

## Materials and methods

### Data sources and processing methods

From The Cancer Genome Atlas (TCGA) database (https://tcga-data.nci.nih.gov/tcga/), we obtained the transcriptome and clinical data from 551 LUSC samples (502 cancer samples and 49 normal samples) on November 18, 2021. After excluding patients for whom there was no survival information, the remaining 495 patients with complete survival information comprised the TCGA-LUSC cohort and were equally randomized into two groups: a training cohort (*n* = 248) and a test cohort (*n* = 247). Based on the information of the patients in the training set, we screened the most valuable lncRNAs, constructed a prognostic signature, and then tested the prognostic signature using the information of the patients in the test set and the whole TCGA-LUSC set to assess the generalizability of the prognostic signature was generalizable. The information of the included patients is listed in Table 1. Fifty-two fever-related genes (PRGs) were obtained by searching the available review literature [17, 18]. All R packages used are based on version 4.1.2 of the R software.

**Table 1:** Clinical information of 495 LUSC samples in the TCGA database

Feature	Train cohort (*n* = 248)		Test cohort (*n* = 247)		Entire TCGA-LUSC cohort (*n* = 495)	
	**n**	**%**	**n**	**%**	**n**	**%**
**Age**						
<=65	88	35.48	101	40.89	189	38.18
>65	158	63.71	142	57.49	300	60.61
Unknow	2	0.81	4	1.62	6	1.21
**Status**						
Alive	140	56.45	145	58.70	285	57.58
Dead	108	43.55	102	41.30	210	42.42
**Gender**		0.00				
Female	59	23.79	70	28.34	129	26.06
Male	189	76.21	177	71.66	366	73.94
**Stage**				0.00		
Stage I	130	52.42	111	44.94	241	48.69
Stage II	75	30.24	85	34.41	160	32.32
Stage III	37	14.92	46	18.62	83	16.77
Stage VI	4	1.61	3	1.21	7	1.41
Unknow	2	0.81	2	0.81	4	0.81
**T stage**						
T1	57	22.98	57	23.08	114	23.03
T2	146	58.87	142	57.49	288	58.18
T3	33	13.31	37	14.98	70	14.14
T4	12	4.84	11	4.45	23	4.65
**M stage**						
M0	206	83.06	201	81.38	407	82.22
M1	4	1.61	3	1.21	7	1.41
Unknow	38	15.32	43	17.41	81	16.36
**N stage**						
N0	168	67.74	148	59.92	316	63.84
N1	55	22.18	73	29.55	128	25.86
N2	20	8.06	20	8.10	40	8.08
N3	1	0.40	4	1.62	5	1.01
Unknow	4	1.61	2	0.81	6	1.21

### Identification of differentially expressed PRlncRNAs

Based on PRGs, we performed a Pearson correlation analysis to identify PRlncRNAs, and results with a *p* value < 0.001 and a correlation coefficient |*R*^2^| > 0.3 were considered reliable. By comparing the differences in RNA expression levels between 49 normal samples and 502 tumor samples, we identified differentially expressed PRGs (DE-PRGs) and differentially expressed PRlncRNAs (DE-PRlncRNAs) by using the “edgeR” package with the criteria of *p* < 0.05 and |log2FC|≥1.

### Construction of the PRlncRNA prognostic signature

Using the “glmnet” package in R, we first screened PRlncRNAs with potential predictive value by univariate Cox regression. We then reduced overfitting and increased the signature stability by LASSO (least absolute shrinkage and selection operator) regression. Moreover, we identified PRlncRNAs by multivariate Cox regression to develop a signature and calculated risk scores by combining the expression of PRlncRNAs and their risk coefficients as follows: $\text{R}\text{i}\text{s}\text{k} \text{S}\text{c}\text{o}\text{r}\text{e}=\sum \text{X}\text{i}\times \text{Y}\text{i}$(in the formula, “Xi” denotes the expression of lncRNA “i”, and “Yi” denotes the coefficient of lncRNA “i”).

### Evaluation of the prognostic value of the signature

We separated patients into high-risk and low-risk groups based on the median values of risk scores derived from the signature for all the patients in each cohort. Using the “survival” and “survminer” packages in R, the overall survival (OS) of the two risk groups was compared by Kaplan–Meier (K-M) analysis. In addition, we obtained the corresponding curve by a receiver operating characteristic (ROC) analysis and calculated the area under the curve (AUC) using the “time ROC” package [19].

### Assessment of the clinical significance of the signature

Through both univariate and multivariate Cox regression analyses, clinicopathological features and risk scores were tested with respect to their value as independent prognostic predictors for LUSC patients [20]. Decision curve analysis (DCA) and ROC analysis were performed to assess the risk signature and other risk indicators. The “scatterplot3D” package was used to evaluate the distribution of patients with different risk scores by principal component analysis (PCA) [21]. We also derived risk scores for patients of various sexes (male and female), ages (65 and > 65), and stages (stages I-II and III-IV). We also created a prognostic nomogram by integrating all risk factors to provide the OS at 1, 3, and 5 years for LUSC cases.

### Stratification analysis

Clinical information was obtained from the TCGA database, and we then grouped the patients according to clinicopathological characteristics and compared the OS of patients in each group by stratification analysis, with the aim to determine the scope of the applicability of the signature.

### Functional analysis

We screened gene sets with variations between the two risk groups. Through a functional pathway enrichment analysis on these gene sets, we then identified and reserved the pathways with NOM *P* < 0.05 and FDR < 0.25, which were considered meaningful [22].

### Immune microenvironment analysis

We used seven different methods (TIMER, QUENTISED, XCELL, CIBERSPOT, CIBERSPOT-ABS, MCPCOUNTER, and EPIC) to calculate the levels of immune cell infiltration between the two risk groups in the tumor immune microenvironment, and the results are shown as heatmaps. We also explored the correlation between immune infiltrating cells and risk scores using the CIBERSPOT method [23]. To further explore the roles of PRlncRNAs and immune function, we also selected 14 immune function pathways and scored them according to the expression of genes involved in these pathways. Depending on these scores, the differences in immune function between the two risk groups were compared.

### Correlation analysis of risk scores with immunotherapy and chemotherapy

To investigate the relationship between risk scores and immunotherapy, we assessed the likelihood of immunotherapy in the high- and low-risk groups based on the Tumor Immune Dysfunction and Exclusion (TIDE) algorithm [24]. In addition, we collected 48 immune checkpoint genes and compare their expression levels between the two risk groups, and *P* < 0.05 was considered to indicate reliability. In addition, we calculated the lower half inhibitory concentration (IC50) of widely used chemotherapeutic drugs using the pRRophetic package and thus compared the difference in sensitivity to different chemotherapeutic drugs between the two risk groups.

## Results

### Identification of PRlncRNAs with predictive value and construction of a signature

**Fig. 1:**
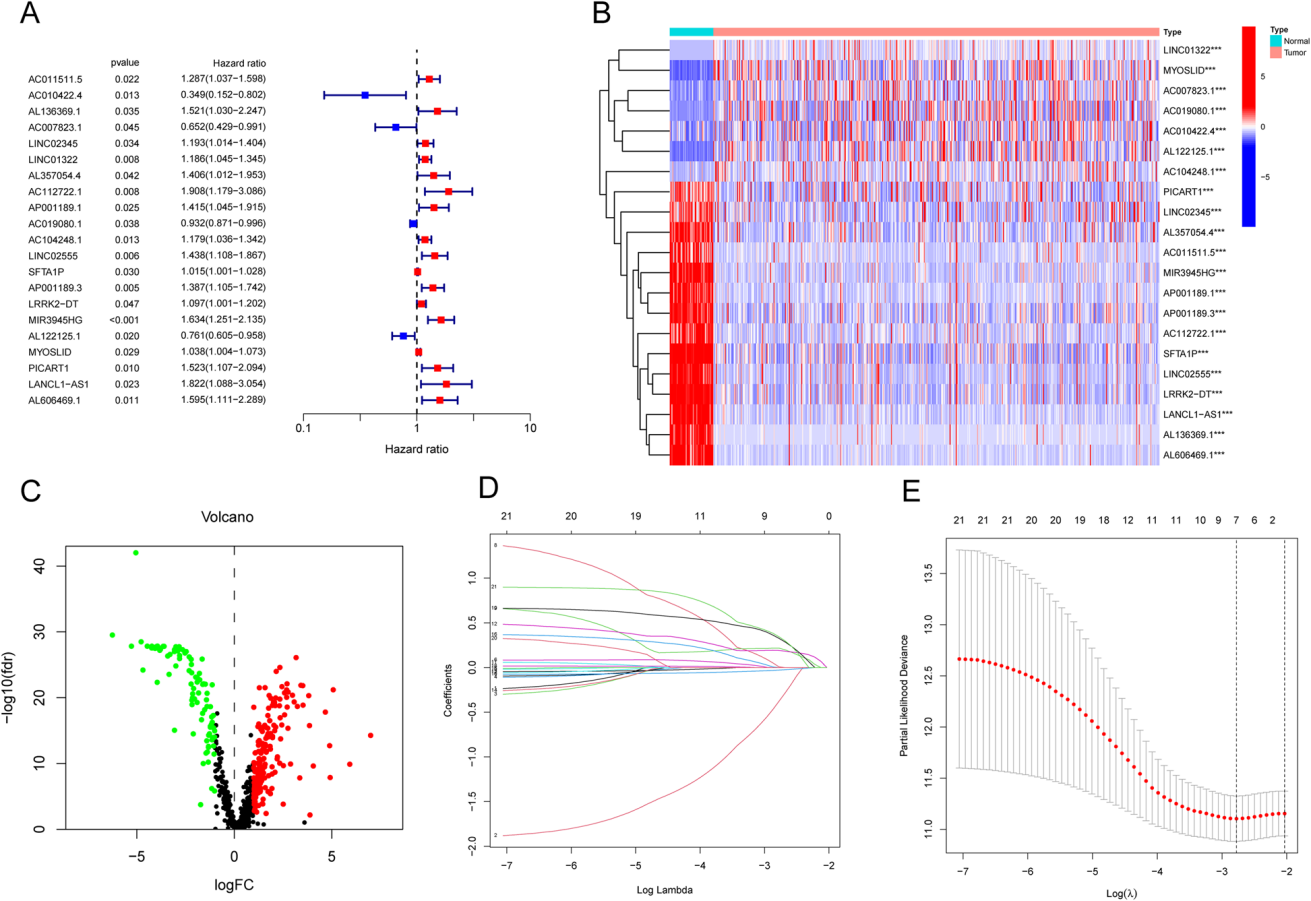
Determining pyroptosis-related lncRNAs (PRlncRNAs) with predictive value. **A** Univariate Cox regression analysis of 21 PRlncRNAs connected with the OS. **B** Heatmap for expression levels of 21 PRlncRNAs associated with prognostic. **C** Volcano plot of DE-PRlncRNAs between normal samples and LUSC samples. **D**, **E** LASSO regression analysis

The overall idea of our study is shown in the flow chart (Fig. S1). First, 743 PRlncRNAs were screened through a Pearson correlation analysis based on the 52 identified PRGs. Among these lncRNAs, 277 DE-PRlncRNAs were identified by the “edgeR” package (Fig. 1A). To further explore the connection between each PRlncRNA and the OS status of LUSC patients, we randomly divided the 495 samples equally into two cohorts. The expression levels of 21 PRlncRNAs are shown in the heatmap (Fig. 1B). As illustrated in the forest plot, in the training cohort, 21 PRlncRNAs connected to the OS of LUSC patients were selected from among the DE-PRlncRNAs (Fig. 1C). Information on these PRlncRNAs is shown in Table S1. A LASSO analysis screened 7 PRlncRNAs (AC010422.4, AC112722.1, AP001189.1, AC019080.1, LINC02555, PICART1, and AL606469.1) with stable prognostic value (Fig. 1D and E). Multivariate Cox regression, identified five PRlncRNAs (AP001189.1, PICART1, LINC02555, AC010422.4, and AL606469.1) were identified to establish a signature. The formula derived from the signature was as follows: risk score =​ (AC019080.1×-0.0877881329685924) + ​(PICART1 × 0.660046397707493) + ​(LINC02555 × 0.419674570168194) + ​(AC010422.4×-1.42076072723105) +(AL606469.1 × 0.737100982354032)​.

### Assessment of the prognostic value of the prognostic signature

**Fig. 2:**
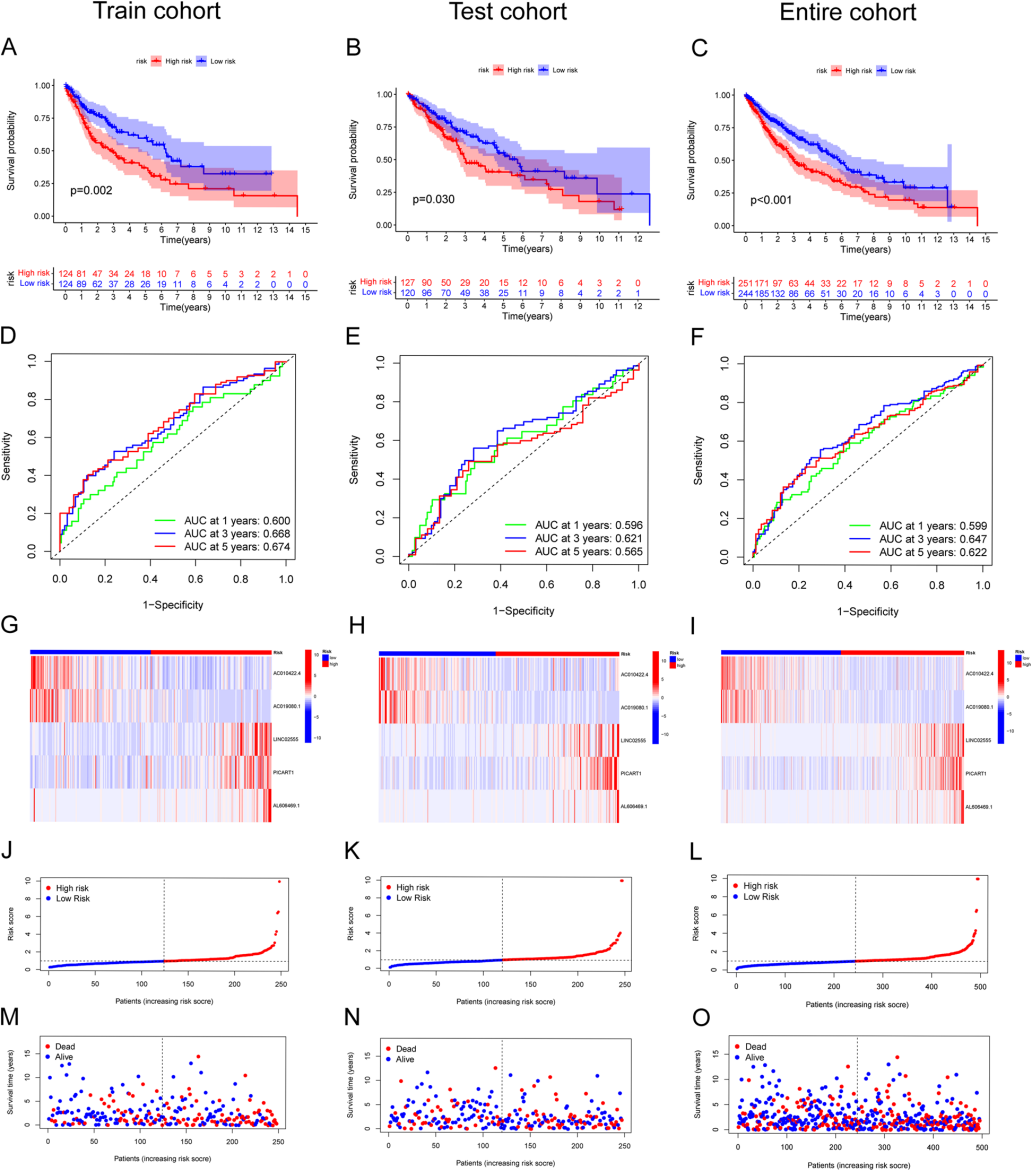
Assessment of the prognostic value of the signature in the training cohort, test cohort, and TCGA-LUSC cohort. **A**-**C** Kaplan–Meier (**K**-**M**) analysis and **D**-**F** Time-dependent ROC curves to compare the survival of high-risk group and low-risk group. **G**-**I** Heatmap for expression levels of 5 PRlncRNAs involved in the signature. **J**-**L** Risk curve for risk scores and **M**-**O** Scatterplot for the survival status of each patient

**Fig. 3:**
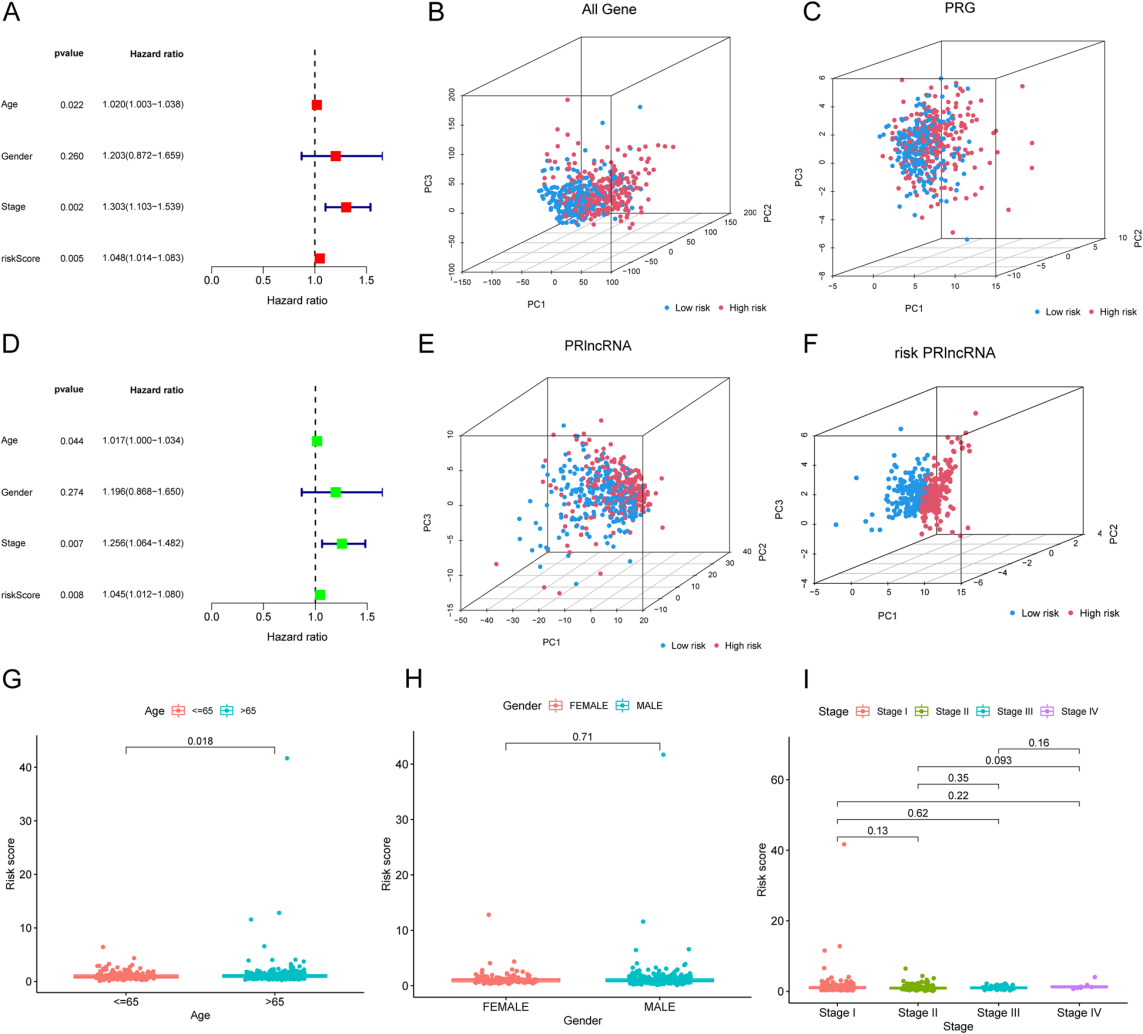
PCA and independent prognostic analysis of the signature. **A** Univariate and **D** multivariate Cox regression analysis to investigate the connection between OS and clinical factors including risk score. Principal component analysis (PCA) based on **B** all genes, **C** all lncRNAs, **E** pyroptosis-related lncRNAs, and **F** risk signature. Correlation analysis of risk signature with **G** age, **H** gender, and **I** state

The patients in each cohort were ranked and then divided into two risk groups (high-risk group and low-risk group) based on the risk scores. The clinical information on the two risk groups is shown in Table 2. In the survival analysis of patients in the two risk groups, the K–M curves revealed a difference in survival between the two groups in the training cohort (Fig. 2A, *p* = 0.002), test cohort (Fig. 2B, *p* = 0.030), and entire cohort (Fig. 2 C, *p* < 0.001). In the three cohorts, the high-risk group in the three cohorts had poorer survival, and the risk scores showed positive correlations with their prognoses. Moreover, the results from the ROC curve analysis results showed that the areas under the curve (AUCs) at 1, 3, and 5 years were 0.600, 0.668, and 0.674, respectively, in the training cohort (Fig. 2D); 0.596, 0.621, and 0.565, respectively, in the test cohort (Fig. 2E); and 0.599, 0.647, and 0.622, respectively, in the TCGA-LUSC cohort (Fig. 2F), which implies that our signature has good value as a prognostic predictor for LUSC patients. The expression of the five PRlncRNAs included in the signature is shown in the heatmap (Fig. 2G-I). The risk curves depicted the risk scores of the patients in the two risk groups (Fig. 2J-L). The risk survival status plot also demonstrated that high-risk patients were mostly concentrated at the bottom (Fig. 2M-O), which revealed the negative correlation of the risk score with the survival time of the patients. The PCA results revealed that using the whole gene, all PRGs, or all PRlncRNAs was not effective in distinguishing patients with different prognoses (Fig. 3B C, and E), whereas using risk signature-related PRlncRNAs was effective for distinguishing patients with different prognoses (Fig. 3F).

**Table 2:** Clinical features of LUSC patients in two risk groups

Feature	High-risk group (*n* = 251)		Low-risk group (*n* = 244)	
	**n**	**%**	**n**	**%**
**Age**				
<=65	86	34.26	103	42.21
>65	163	64.94	137	56.15
Unknow	2	0.80	4	1.64
**Status**				
Alive	128	51.00	157	64.34
Dead	123	49.00	86	35.25
Gender				
Female	71	28.29	58	23.77
Male	180	71.71	186	76.23
**Stage**				
Stage I	127	50.60	114	46.72
Stage II	75	29.88	85	34.84
Stage III	42	16.73	41	16.80
Stage VI	5	1.99	2	0.82
Unknow	2	0.80	2	0.82
**T stage**				
T1	59	23.51	55	22.54
T2	146	58.17	142	58.20
T3	36	14.34	34	13.93
T4	10	3.98	13	5.33
**M stage**				
M0	207	82.47	200	81.97
M1	5	1.99	2	0.82
Unknow	39	15.54	42	17.21
**N stage**				
N0	171	68.13	145	59.43
N1	53	21.12	75	30.74
N2	21	8.37	19	7.79
N3	3	1.20	2	0.82
Unknow	3	1.20	3	1.23

### Correlation analysis of the prognostic signature and clinical features

**Fig. 4:**
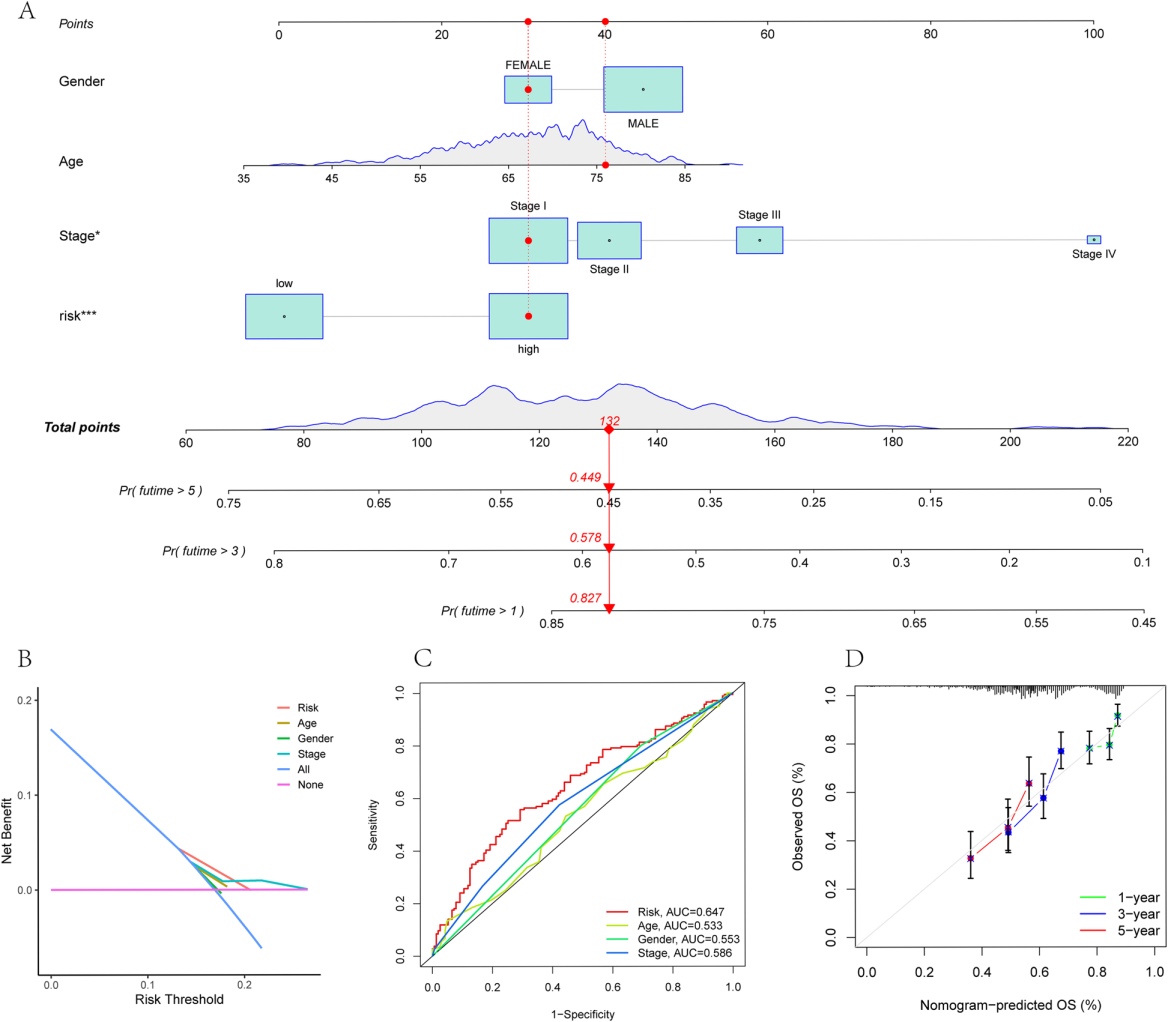
Development of a nomogram by combining all risk factors. **A** Nomogram containing age, gender, stage, and risk score to predict survival. **B** DCA analysis of clinical features including risk signature. **C** ROC analysis of predicting prognosis based on different prognostic signatures. **D** Calibration curve to test the nomogram

Univariate and multivariate Cox regression analyses were performed using the entire TCGA cohort (Table S2), which revealed that the risk score (*p* = 0.005), age (*p* = 0.022), and state (*p* = 0.002) affected the OS of LUSC patients (Fig. 3A). Moreover, the results also confirmed the independent predictive value of the risk score (*p* = 0.008), state (*p* = 0.007) and age (*p* = 0.044) (Fig. 3D). According to the correlations of the risk scores with other risk factors, age (*p* = 0.015) was significantly correlated with the risk scores, whereas gender and stage were not significantly associated with risk scores (Fig. 3G-I). In addition, a nomogram of the risk scores and other valuable predictors was constructed to more accurately predict the 1-, 3- and 5-year survival of the patients (Fig. 4A). The DCA curve indicated that the risk score had better predictive value than other risk factors (Fig. 4B). ROC analysis also demonstrated that the AUC for age (0.533), gender (0.553), and stage (0.586) was less than the AUC for the risk score (0.647) (Fig. 4C). The calibration curves showed good consistency between the predicted and observed OS at 1, 3, and 5 years, which proved the accuracy of the nomogram in predicting survival (Fig. 4D).

### 5-PRlncRNAs signature is more suitable for early-stage LUSC patients

The clinical features of the patients in the two risk groups and the five PRlncRNAs included in the signature are shown in the heatmap (Fig. S2A). To determine the scope of the applicability of the signature, we performed stratification analysis of the patients according to the following subgroups: female and male; age ≤ 65 and age > 65; tumor stage I-II and stage III-IV; T1–2 and T3–4; M0 and M1; N0 and N1–3 (Fig. S2B-M). As the results revealed, the signature had good predictive power for men of all ages, particularly for patients with early-stage LUSC.

### Functional pathway enrichment analysis of different gene sets in the high-risk and low-risk groups

**Fig. 5:**
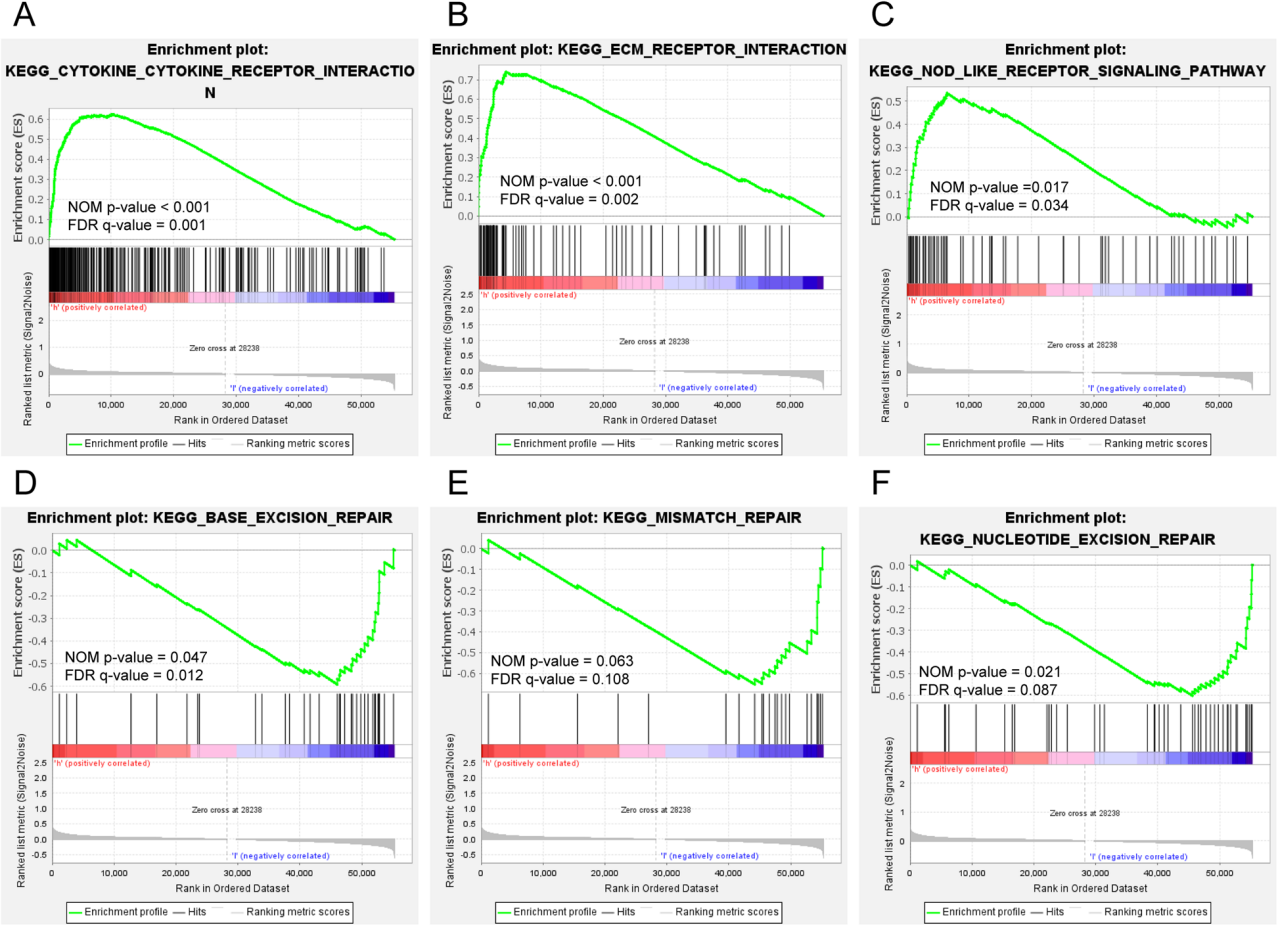
Functional analysis. Valuable functional pathways in **A**-**C** high-risk groups and **D**-**F** low-risk groups

To investigate the functional pathways related to differentially expressed gene sets between the two risk groups, we applied GSEA, which showed that the main enriched pathways related to the set of genes more highly expressed in the high-risk group were as follows: “CYTOKINE receptor interaction” (Fig. 5A, NOM *P* < 0.001, FDR = 0.001), “ECM receptor interaction” (Fig. 5B, NOM *P* < 0.001, FDR = 0.002) and “NOD-like signaling pathway” (Fig. 5C, NOM *P* = 0.017, FDR = 0.034). The main enriched pathways related to the set of genes more highly expressed in the low-risk group were as follows: “base excision repair” (Fig. 5D, NOM *P* = 0.047, FDR = 0.012), “mismatch repair” (Fig. 5E, NOM *P* = 0.063, FDR = 0.108) and “nucleotide excision repair” (Fig. 5 F, NOM *P* = 0.021, FDR = 0.087).

### Landscape of the immune cell infiltration and immune functional pathways enrichment

**Fig. 6:**
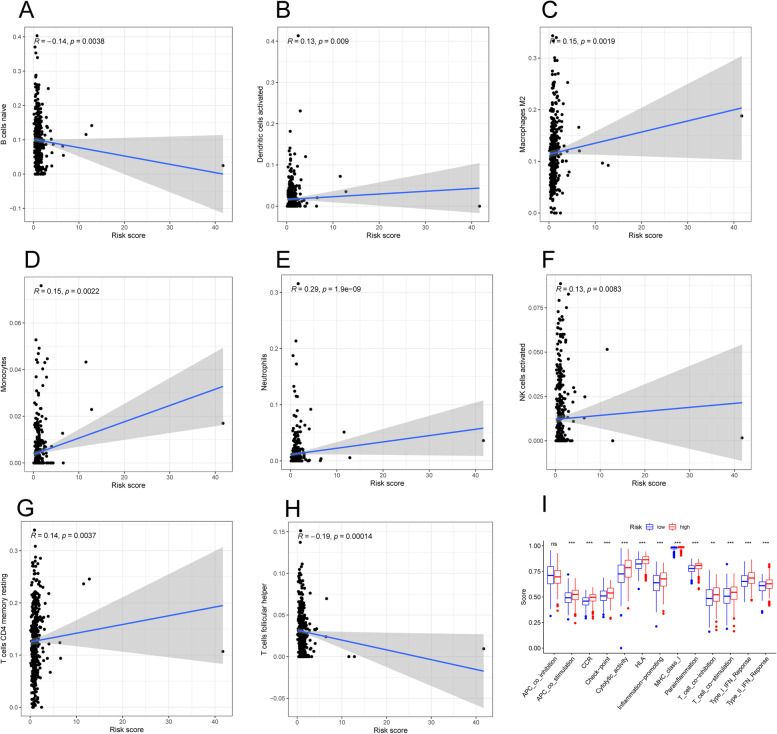
Correlation analysis of tumor immune microenvironment. **A**-**H** The relationship between 8 different types of immune cells and risk scores according to the results of CIBERSPOT. **I** Boxplots for immune functions scores between two risk groups

The heatmap showed that most immune cells exhibited more significant infiltration in the high-risk group (Fig. S3). Negative connections between risk scores and the infiltration levels of naïve B cells (Fig. 6A, *p* = 0.0038) and follicular helper T cells (Fig. 6H, *p* = 0.00014) was revealed by the CIBERSORT method. Moreover, there was a connection was detected between the risk scores and the infiltration level of activated dendritic cells (Fig. 6B, *p* = 0.009), M2 macrophages (Fig. 6C, *p* = 0.0019), monocytes (Fig. 6D, *p* = 0.0022), neutrophils (Fig. 6E, *p* = 1.9e-0.9), activated NK cells (Fig. 6F, *p* = 0.0083) and resting memory CD4 + T cells (Fig. 6G, p = 0.0037). An immune function analysis suggested that higher risk scores were strongly correlated with 14 immune function pathways (Fig. 6I).

### Risk scores serve as a predictor of sensitivity to immunotherapy and chemotherapy

According to the TIDE algorithm, patients in the high-risk group are more prone to immune escape, whereas patients in the low-risk group may be more sensitive to immunotherapy, which indicates that the risk score can be used as a predictor of sensitivity to immunotherapy (Fig. S4A). The results from the immune checkpoint gene expression analysis showed that 23 immune checkpoint genes were highly expressed in the high-risk group and that 8 immune checkpoint genes were highly expressed in the low-risk group, and all these genes could be used as immunotherapy targets (Fig. S4B). The results from the drug sensitivity analysis showed that the low-risk group was more sensitive to A.443,654 and ATRA (Fig. S4C and D, *p* < 0.001), whereas the high-risk group was more sensitive to A.770,041, AMG.706, AP.24,534, A S601245, AUY922, AZ628 and AZD.0530 (Fig. S4E-K, *p* < 0.001), which suggested that the prognostic signature could predict sensitivity to chemotherapy.

## Discussion

LUSC is a common tumor type and remains a challenge that remains to be overcome [25]. The lack of improved tools for the prediction of prognoses and better immunotherapy options makes the prognosis of LUSC patients poor [5]. Pyroptosis, as a novel form of programmed death closely related to inflammatory factors and gasdermin (GSDM) proteins, provides the possibility of solving these challenges [26]. Pyroptosis is a double-edged sword for tumors. On the one hand, it has been shown that differential expression of gasdermin (GSDM) proteins in cells can cause the hypermethylation of pyroptosis genes, and thus allowing the transformation of normal cells into tumor cells [27]. On the other hand, it has also been shown that the activation of gasdermin (GSDM) protein-mediated pyroptosis could be a new target therapy for a variety of tumors, including LUSC [28]. lncRNAs, as important factors affecting the treatment and prognoses of tumor patients, are considered new biomarkers and therapeutic targets for LUSC. Some studies have confirmed the connection between lncRNAs and pyroptosis, but their specific roles in pyroptosis remain unclear [14]. Therefore, there is an urgent need to investigate the relationships between PRlncRNAs and LUSC.

In this study, we divided 495 LUSC cases with complete information into two cohorts. By analyzing the training cohort, we identified five PRlncRNAs (AP001189.1, PICART1, LINC02555, AC010422.4, and AL606469.1) to develop a prognostic signature for patients with LUSC. Among the five PRlncRNAs included in the signature, the lncRNA PICART1 is closely associated with the suppression of lung cancer cell proliferation by targeting the AKT1 and JAK2/STAT3 signaling pathways [29, 30]. LINC02555 has been confirmed to strongly affect the prognoses of patients with LUSC [31]. Details of the other three included PRlncRNAs are still lacking. In addition, we validated the predictive power and independent predictive value of the five-PRlncRNA signature and constructed a nomogram.

To explore the enrichment of gene set functional pathways that differed between the two risk groups, a GSEA was performed between the two groups. According to the results, pathways associated with tumor development and pyroptosis were mainly enriched in the high-risk group, whereas in the low-risk group, several pathways of molecular repair were mainly enriched in the low-risk group. As a group of receptors, the NOD-like receptor (NLR) family mediates cell injury pattern recognition and is potentially associated with programmed death and immunity. Previous studies identified a multiprotein complex formed by NLR activated caspase-1 that cleaves cleaved gasdermin D and triggers pyroptosis [32]. Recently, Peng Liu et al. revealed that NOD-like receptor signaling plays an important role in inflammation-related cancers, and this finding provides a new direction for immunotherapy development [33]. The “CYTOKINE receptor interaction” pathway, as well as the “ECM receptor interaction” pathway, have been shown to be closely linked to the processes of tumor cell growth and invasion [34, 35]. On the one hand, gene sets enriched in “repair pathways”, on the one hand, reduce the cell mutation rate and tumorigenesis, and on the other hand, these gene sets make it more difficult for tumor cells to be affected by therapeutic measures. For instance, Grundy et al. revealed that the base excision repair system enhances tumor cell resistance to oxidative stress and thus reduces the efficacy of tumor radiotherapy and chemotherapy [36]. Anne et al. revealed that nucleotide excision repair (NER) activity significantly affects the sensitivity of DNA damage factors and thus affects the efficacy of platinum-based chemotherapeutic agents [37]. The mismatch repair system has also been shown to affect satellite instability and reduce the effectiveness of immunotherapy [38].

Changes in the tumor microenvironment affect the development of tumors, which manifesting both antitumor and protumor properties [39]. Our findings suggest that the majority of immune cell infiltration levels, including those of M2 macrophages, monocytes, and CD4 + T cells, increase with increases in the patient risk scores. It has been proven that tumor-associated macrophages are important protumor components of the tumor microenvironment [40]. Additionally, sufficient studies have demonstrated that CD4 + T cell infiltration is related to the efficacy of tumor treatment with immunotherapy and that more abundant CD4 + T cell infiltration usually means that a tumor is “hot” to immunotherapy [41]. Immune checkpoint inhibitors can provide a direction idea for immunotherapy by consistently causing tumor progression due to immune escape. Immune checkpoint inhibitors, as emerging chemotherapy agents, have been used to benefit patients with a variety of cancers [41]. There is growing evidence of the feasibility of immunotherapy in LUSC, but effective immune checkpoint-related genes in LUSC still lack sufficient studies [4]. This study demonstrated that the signature can be used as a factor to predict the sensitivity of LUSC patients to immunotherapy and chemotherapy. We also identified 32 immune checkpoint genes that were differentially expressed in the high- and low-risk groups, which can suggest ideas for the study of new targets for immunotherapy.

To better evaluate the scientific nature of the signature, we collected other articles on constructing signatures that can be compared with ours. Although no other study has constructed a PRlncRNA signature to predict the prognosis of LUSC patients, we noted that Huang et al. constructed a PRlncRNA signature to predict the prognosis of LUAD patients [42]. Their processes for constructing and verifying the model, analyzing the immune microenvironment and evaluating the correlation between the model and immunotherapy were similar to ours. However, we also integrated the signature and traditional risk factors and developed a nomogram to improve the intuitiveness and accuracy of the prediction. In addition, we noted that Weng et al. constructed a m6A gene-related model to predict the prognosis of LUSC patients [43]. The utility of their signature was similar to ours, but in contrast, we performed internal validation during signature construction, and the AUC of our signature (AUC = 0.647) was higher than that constructed in the previous study (AUC = 0.572), which indicated that our signature was more feasible. In addition, we found some predictive signatures for other types of cancer that are important to us. Cao et al. introduced more risk factors, such as the smoking history, when evaluating the correlation between the model and traditional risk factors, which also had a significant impact on lung squamous cell carcinoma [44]. Some articles mentioned the significance of weighted gene coexpression network analysis (WGCNA) for the model, which has a significant impact on our subsequent research [45].

There are still some shortcomings in our work. First, experiments validating the expression and functions of PRlncRNAs involved in the signature are lacking but will be performed in the future by our laboratory. Second, the limited number of normal samples in the TCGA database may have influenced the results of the differential analysis. Third, more work is needed to explore how pyroptosis regulates the tumor immune microenvironment in LUSC. Fourth, most of the data in TCGA are from North America and are predominantly from white patients, which limits the applicability of the signature.

## Conclusions

In conclusion, we first screened five PRlncRNAs and accordingly derived a prognostic signature suitable for patients with early-stage LUSC. We also tested the prognostic and clinical value of the constructed signature and combined this signature with other clinicopathological features to create a nomogram that could more accurately predict the survival status of patients. Furthermore, our study suggests that PRlncRNAs are closely associated with the LUSC tumor immune microenvironment and may be potential targets for immunotherapy.

## Supplementary Information

**Additional file 1.**

## Data Availability

The data sets used and/or analysed during the current study are available from the corresponding author on reasonable request.

## Abbreviations

DCA: Decision curve analysis
FDR: False discovery rate
GSEA: Gene set enrichment analysis
H: high
HR: Hazard ratios
IC50: the lower half inhibitory concentration
K-M survival analysis: Kaplan-Meier survival analysis
L: low
LC: Lung cancer
lncRNA: Long non-coding RNA
LUAD: Lung adenocarcinoma carcinoma
LUSC: Lung squamous cell carcinoma
NLR: NOD-like receptor
OS: Overall survival
PCA: Principal component analysis
PRG: Pyroptosis-related gene
PRlncRNA: Pyroptosis-related long non-coding RNA
ROC analysis: Time receiver-operating characteristic analysis
TCGA: The Cancer Genome Atlas
TIDE: Tumor Immune Dysfunction and Exclusion
WGCNA: Weighted gene Co-expression Network analysis
