# Supplementary material for: A novel pyroptosis-related lncRNA prognostic signature associated with the immune microenvironment in lung squamous cell carcinoma
Source: BMC Cancer. 2022 Jun 23;22:694. doi: 10.1186/s12885-022-09790-z (PMC9229145; doi:10.1186/s12885-022-09790-z)
Supplement: Supplementary file 1 — Additional file 1. [file 12885_2022_9790_MOESM1_ESM.pdf]

Supplementary information

Figure S1 Flowchart.

Figure S2 Subgroup validation based on clinical features. (A) Heatmap for expression levels and correlation of risk signature with clinical factors. (B-M) Stratification analysis of survival state in the clinical groups with different clinical features.

Figure S3 Heatmap for immune cell infiltration level in the two risk groups based on different analytic methods.

Figure S4 Correlation analysis of risk scores with immunotherapy and chemotherapy sensitivity. (A) Correlation analysis of risk scores with immunotherapy based on TIDE algorithm. (B) Immune checkpoint genes expression level between two risk groups. (C-K) The relationship between risk scores and IC50 of different chemotherapeutic drugs.

Table S1 21 PRlncRNAs connected with the survival state of the LUSC patients.

Table S2 Univariate and multivariate Cox regression analysis of risk factors.

Figure S1 Flowchart.

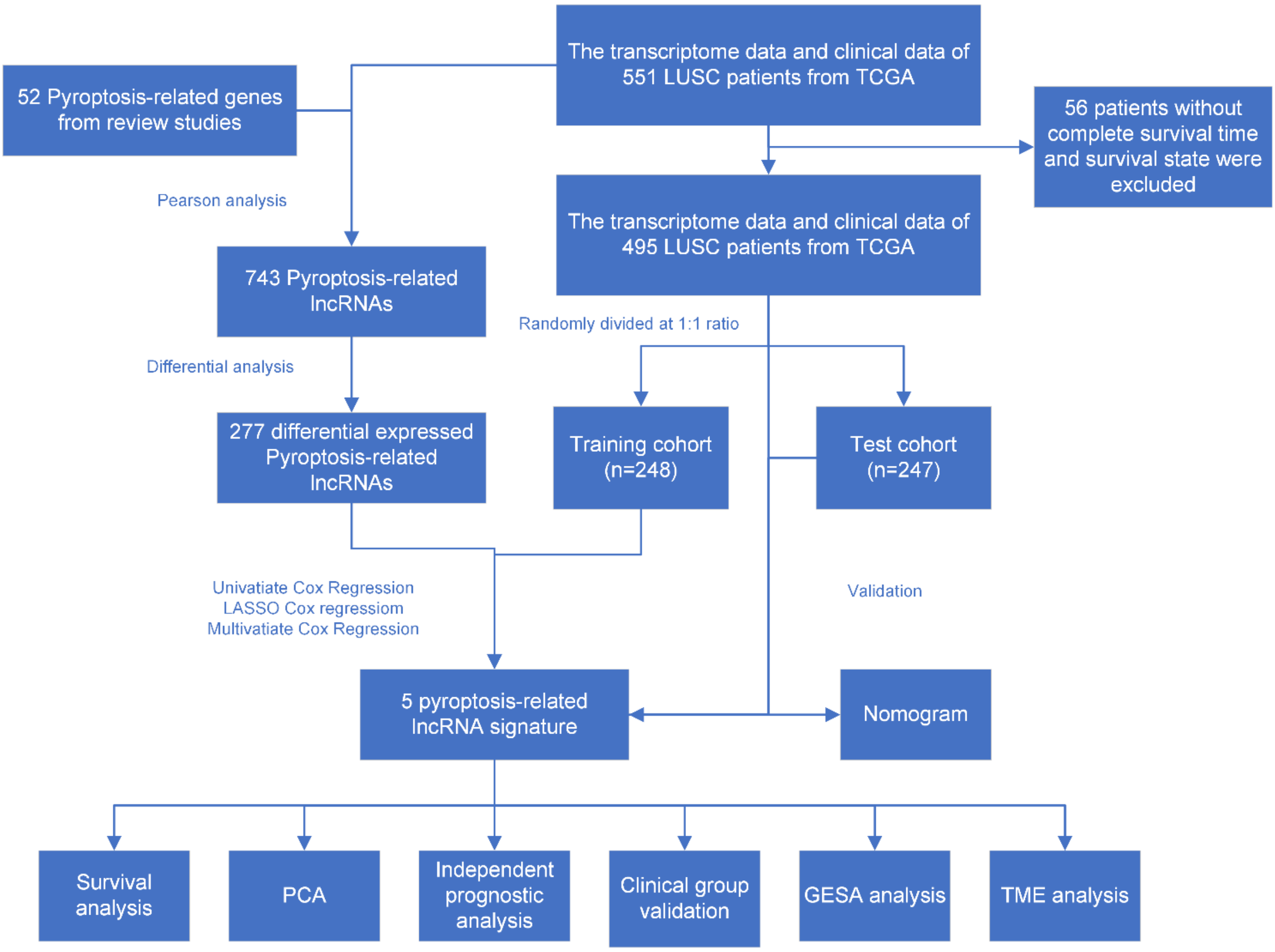

Figure S2 Subgroup validation based on clinical features. (A) Heatmap for expression levels and correlation of risk signature with clinical factors. (B-M) Stratification analysis of survival state in the clinical groups with different clinical features.

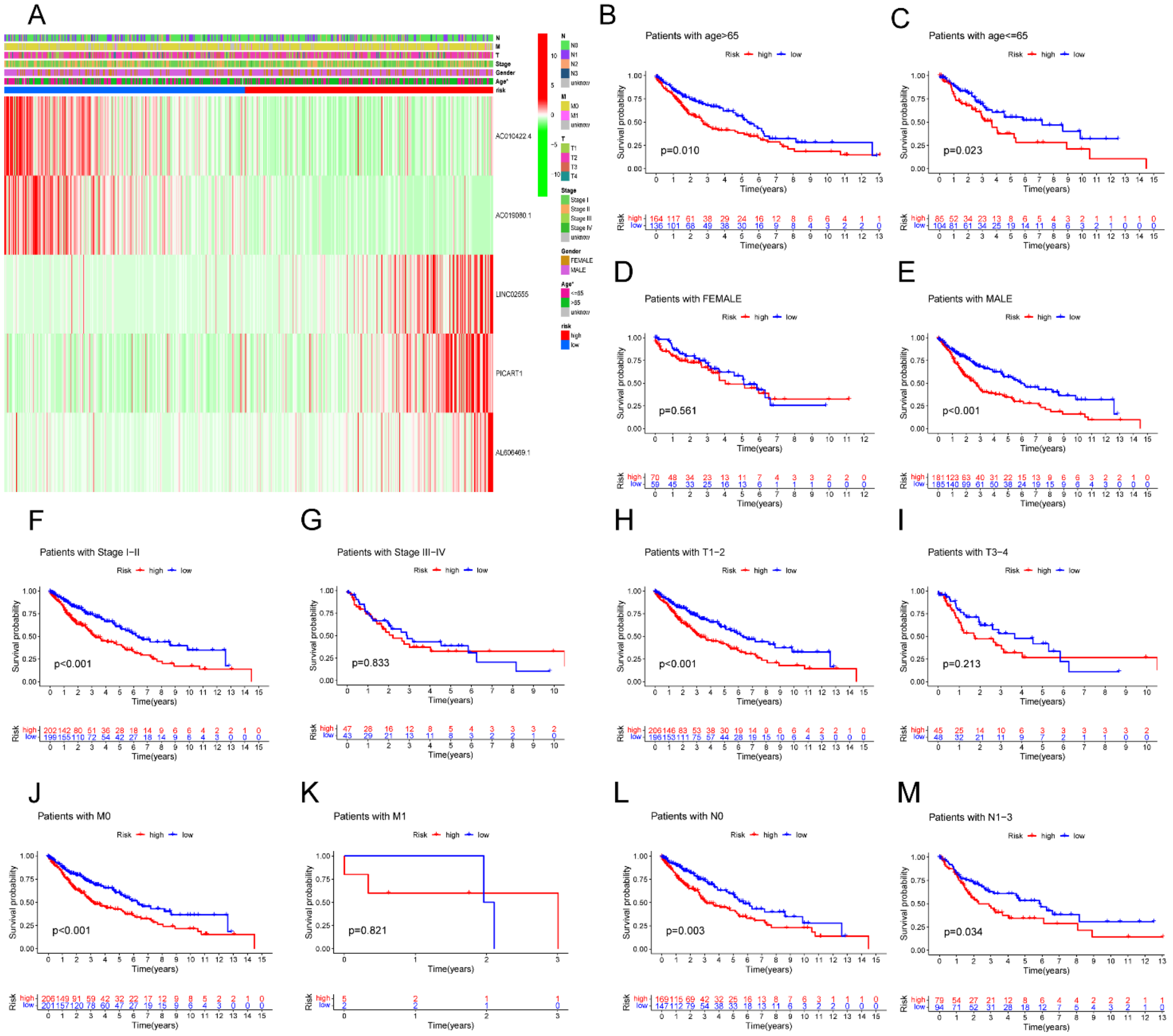

Figure S3 Heatmap for immune cell infiltration level in the two risk groups based on different analytic methods.

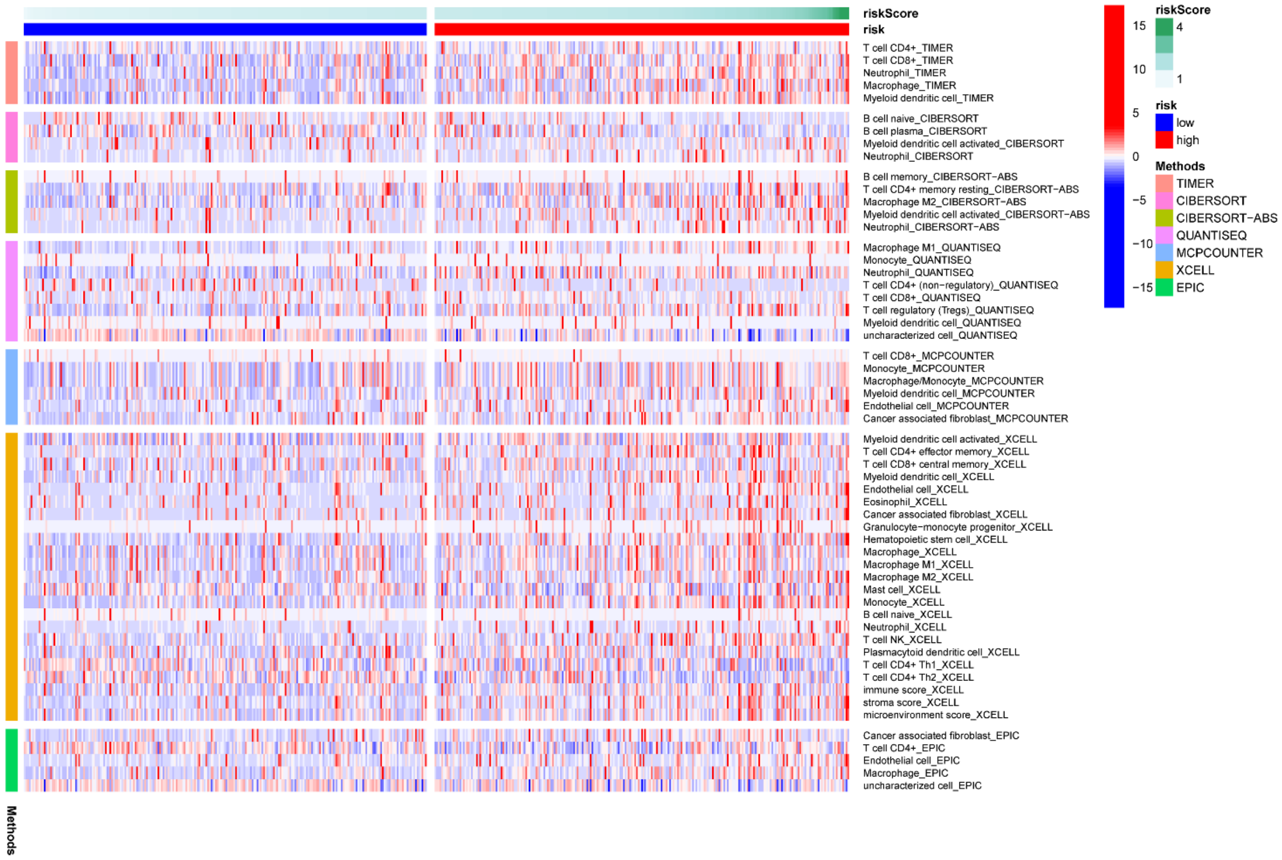

Figure S4 Correlation analysis of risk scores with immunotherapy and chemotherapy sensitivity. (A) Correlation analysis of risk scores with immunotherapy based on TIDE algorithm. (B) Immune checkpoint genes expression level between two risk groups. (C-K) The relationship between risk scores and IC50 of different chemotherapeutic drugs.

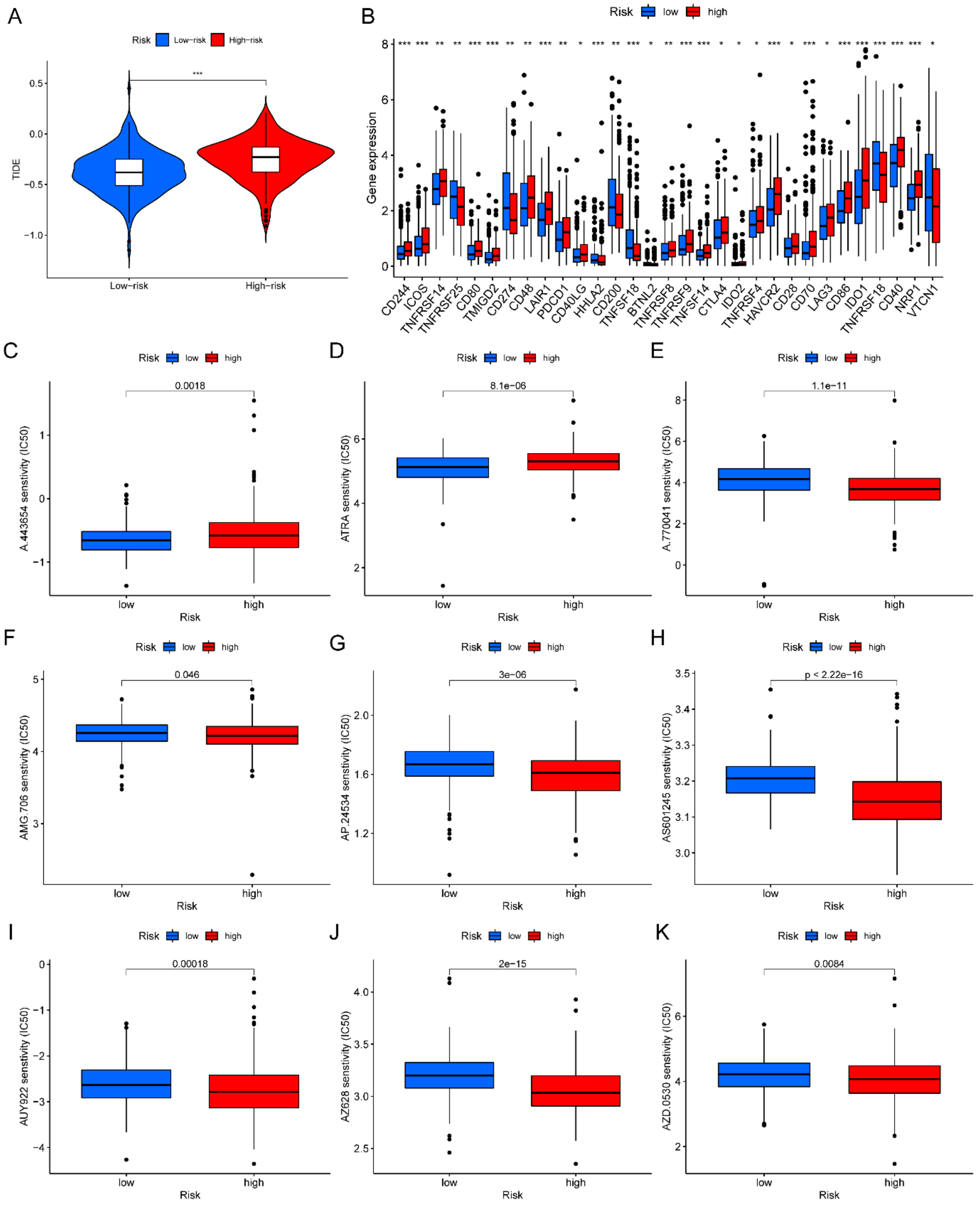

Table S1 21 PRlncRNAs connected with the survival state of the LUSC patients.

Gene	HR	HR (95%L)	HR (95%H)	P-value
AC011511.5	1.29	1.04	1.60	0.02
AC010422.4	0.35	0.15	0.80	0.01
AL136369.1	1.52	1.03	2.25	0.03
AC007823.1	0.65	0.43	0.99	0.05
LINC02345	1.19	1.01	1.40	0.03
LINC01322	1.19	1.05	1.35	0.008
AL357054.4	1.41	1.01	1.95	0.04
AC112722.1	1.91	1.18	3.09	0.008
AP001189.1	1.41	1.05	1.92	0.02
AC019080.1	0.93	0.87	1.00	0.04
AC104248.1	1.18	1.04	1.34	0.01
LINC02555	1.44	1.11	1.87	0.006
SFTA1P	1.01	1.00	1.03	0.03
AP001189.3	1.39	1.11	1.74	0.005
LRRK2-DT	1.10	1.00	1.20	0.05
MIR3945HG	1.63	1.25	2.13	<0.001
AL122125.1	0.76	0.61	0.96	0.02
MYOSLID	1.04	1.00	1.07	0.03
PICART1	1.52	1.11	2.09	0.01
LANCL1-AS1	1.82	1.09	3.05	0.02
AL606469.1	1.59	1.11	2.29	0.01

Abbreviation: H: high; HR: Hazard ratios; L: low; LUSC: Lung squamous carcinoma; OS: Over survival; PRlncRNA: Pyroptosis-related long non-coding RNA.

Table S2 Univariate and multivariate Cox regression analysis of risk factors.

**Item**	**Univariate Cox regression**				**Multivariate Cox regression**			
	**HR**	**HR (95%L)**	**HR (95%H)**	**P-value**	**HR**	**HR (95%L)**	**HR (95%H)**	**P-value**
Age	1.02	1.00	1.03	0.04	1.02	1.00	1.04	0.02
Gender	1.20	0.87	1.65	0.27	1.20	0.87	1.66	0.26
Stage	1.26	1.06	1.48	0.007	1.30	1.10	1.54	0.002
Risk Score	1.05	1.01	1.08	0.008	1.05	1.01	1.08	0.005

Abbreviation: HR: Hazard ratios.
